# Effect of Cefazolin Prophylaxis on Postoperative Infectious Complications in Elective Laparoscopic Cholecystectomy: A Prospective Randomized Study

**DOI:** 10.5812/ircmj.11111

**Published:** 2013-07-05

**Authors:** Emin Turk, Erdal Karagulle, Kivanc Serefhanoglu, Hale Turan, Gokhan Moray

**Affiliations:** 1 Department of Surgery, Baskent University, Ankara, Turkey; 2 Infectious Diseases and Clinical Microbiology, Baskent University, Ankara, Turkey

**Keywords:** Antibiotic Prophylaxis, Surgical Wound Infection, Cholecystectomy, Laparoscopic

## Abstract

**Background:**

In patients with symptomatic cholelithiasis, laparoscopic cholecystectomy (LC) is the standard method of treatment. Laparoscopic cholecystectomy has a low rate of postoperative infections probably owing to smaller wounds and minimal tissue damage compared with the open procedure.

**Objectives:**

This study assessed the effect of cefazolin prophylaxis on postoperative infection in patients undergoing elective laparoscopic cholecystectomy. Additionally, we determined the risk factors of cases with postoperative infection.

**Patients and Methods:**

A total of 753 patients were enrolled in the study. Among these, 206 were excluded from the study. As a result, 547 patients with symptomatic cholelithiasis who underwent elective laparoscopic cholecystectomy were selected for this prospective study. Patients were randomized consecutively and divided into 2 groups: patients in the cefazolin (CEF) group (n = 278) received 1 g of cefazolin and those in the control group (n = 269) received 10 mL of isotonic sodium chloride solution. Patient characteristics and overall surgical outcomes were compared between the groups. All patients were followed for development of postoperative infections.

**Results:**

Postoperative infections occurred in 4 patients in the CEF group and in 2 patients in the control group; no significant difference existed in this regard(P = .44). Risk of infection increased in patients with previous cholecystitis and/or endoscopic retrograde cholangiopancreatography (P < 0.001), patients with ruptured gallbladders, and patients for whom a suction drain was used (respectively, P < 0.001 and P < 0.001).

**Conclusions:**

No correlation existed between cefazolin prophylaxis and postoperative infections in elective laparoscopic cholecystectomy patients. There may be an increased risk of infection in patients with previous cholecystitis or endoscopic retrograde cholangiopancreatography. In addition, there was an increased risk of postoperative infection in patients with gallbladder rupture and suction drain use.

## 1. Background

For patients with symptomatic cholelithiasis, laparoscopic cholecystectomy (LC) is the standard method of treatment. Laparoscopic cholecystectomy has a low rate of postoperative infections probably owing to smaller wounds and minimal tissue damage compared with the open procedure (1). Due to its low infection rate and because the use of prophylactic antibiotics does not further decrease the rate of wound infections or other postoperative infections, many investigators believe that antimicrobial prophylaxis may be unnecessary for LC patients (1-8). However, prophylactic antibiotic use in LC remains popular, and many surgeons believe that it decreases the incidence of postoperative infections (9-11). Cefazolin (a first-generation cephalosporin) has some favorable pharmacokinetic properties, including sufficient distribution to the gallbladder wall and a high concentration in bile. These properties make a single 1-g dose of cefazolin effective for antibacterial prophylaxis in biliary tract procedures (ie, endoscopic retrograde cholangiopancreatography [ERCP] for patients with obstructive jaundice or patients with acute cholecystitis at high risk of bacteria in bile) (12, 13). This study sought to investigate the effect of antibiotic prophylaxis on the occurrence of postoperative infection complications in patients undergoing elective LC.

## 2. Objectives

We aimed to determine the risk factors for postoperative infection.

## 3. Patients and Methods

The study’s hypothesis was that using cefazolin in elective laparoscopic cholecystectomy would result in the development of less postoperative infections during the follow up period. For the calculation of sample size, infection rate in elective laparoscopic cholecystectomy operation was assumed to be 4% (literature search indicated an infection rate of 0.4% to 16.3%). Given the assumption that cefazolin application decreases infection rate by 0.5%, both groups included 280 patients to achieve a power level of 80%. Sample sizes were calculated by using the Minitab statistical package program (Release 14). The study was conducted according to the recommendations of the Declaration of Helsinki on Biomedical Research Involving Human Subjects. This prospective, randomized clinical study was performed at the Baskent University Department of General Surgery after obtaining the approval of the University Ethics Committee (KA09/400-date: 2009/8/15). All patients gave written, informed consents before the operation. This study was planned for patients undergoing elective LC between October 2009 and June 2012. The inclusion criteria were (1) being in ASA I and II categories upon anesthesiologic examination and (2) having symptomatic cholelithiasis. Exclusion criteria were (1) having acute cholecystitis. All patients underwent an ultrasonographic examination of the upper abdomen. Acute cholecystitis was diagnosed based on the following criteria: presence of a hydropic gallbladder, gallbladder wall thickness greater than 3 mm, presence of pericholecystic free fluid, and presence of sonographic Murphy’s sign; (2) antibiotic use within 7 days of the planned LC; (3) an anesthesia category of ASA III or higher; (4) hypersensitivity to cephalosporins or beta-lactams; (5) concomitant choledocholithiasis, intrahepatic duct stones, or gallstone pancreatitis; (6) previous hepatic or biliary surgery; (7) conversion to open cholecystectomy; (8) elevated preoperative white blood cell count (> 12.5 × 103/mL).

**Figure 1.**
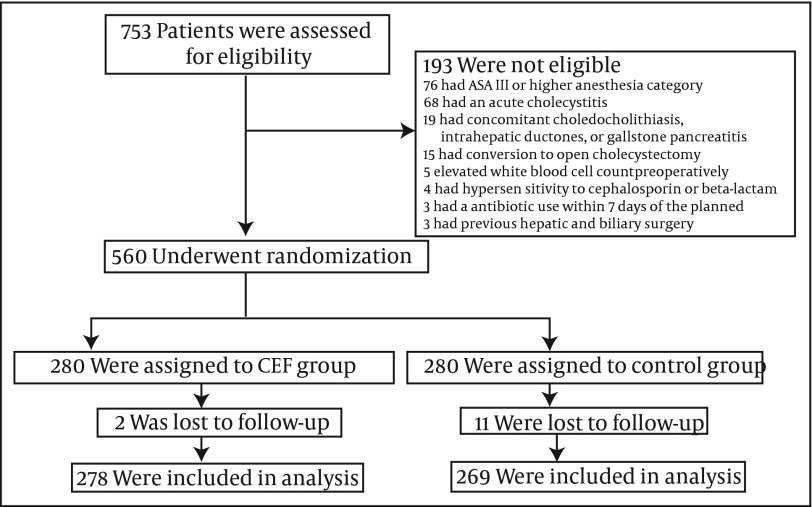
Randomization of the Groups

A total of 753 patients were enrolled in the study. Weight and height of all patients were measured to calculate body mass index. The reasons for exclusion and the number of excluded patients were as follows: 68 had acute cholecystitis, 3 had antibiotic use within 7 days of the planned LC, 76 had an anesthesia category of ASA III or higher, 4 had hypersensitivity to cephalosporins or beta-lactams, 19 had concomitant choledocholithiasis, intrahepatic duct stones, or gallstone pancreatitis, 3 had previous hepatic or biliary surgery, 15 had conversion to open cholecystectomy, 5 had elevated preoperative white blood cell counts. Patients were consecutively and intraoperatively randomized in a 1:1 fashion into two study arms. Emin Turk, MD carried out the randomization and data collection. Thirteen patients did not continue as their own controls. As a result, 547 patients (cefazolin group = 278 subjects, control group = 269 subjects) completed the study (Figure 1). 

All patients included in the study were operated via the laparoscopic cholecystectomy procedure. At the time of anesthesia induction, CEF patients (n = 278) received 1 g of cefazolin and controls (n = 269) received 10 mL of isotonic sodium chloride solution. The patients, anesthesiologists, outcome assessors, data analysts, and other research staff were blinded to the treatment protocol. No additional doses of antibiotics, either intravenous or oral, were administered during or after the surgery for either patient group. All patients were operated using the same laparoscopic device and reusable laparoscopic instruments sterilized with ethylene oxide. Skin was prepared with 10% povidone-iodine solution. Laparoscopic cholecystectomy was done via the 4-trocar standard technique. Gallbladders were extracted through the opening of the trocar-made extraction hole in the subxiphoid region. An endo bag was not used for any patient. Local peritoneal irrigation was performed, and a suction drain was placed in the sub-hepatic area in case of gallbladder rupture and bile spillage. We used 1-0 polyfilament, absorbable, synthetic, braided, polyglactin 910 sutures to close the abdominal fascia. All skin incisions were closed with 2-0 non-absorbable polyfilament silk sutures. The postoperative course was monitored, and incidents such as fever, infection of the trocar site, or intra-abdominal collection of pus were recorded. All patients were examined by an attending surgeon (Erdal Karagulle, MD) 7 to 10 days after the operation and followed for 30 days. The review included a structured interview and a clinical examination. None of the patients received antibiotics after discharge from the hospital.

In the postoperative evaluation, fever, cough, chest pain, abdominal pain, dysuria, and nausea and vomiting were questioned first. All patients underwent physical examination and patients suspected to have an infection were investigated for an infectious focus. Depending on the findings, infectious parameters (leucocyte count, high-sensitivity C-reactive protein), liver function tests (total/direct bilirubin, gamma glutamyl transferase, alkaline phosphatase, aspartate aminotransferase, alanine aminotransferase), urinalysis, and urine culture were ordered. Patients with abnormal results from any of these investigations and/or patients suspected to have an infection clinically underwent a two-sided chest X-Ray, abdominal ultrasonography, and/or thoracic and/or abdominal computed tomography. Postoperative monitorized data and other test results were evaluated simultaneously for the decision. Infectious complications were defined as pyrexia with a body temperature higher than 38°C twice a day (excluding the first postoperative day) and/or a culture positivity of pathogens from infectious sites such as the wound site, urinary or respiratory tract, or the abdominal cavity. If bacteria were found in the culture, sensitivity to antimicrobial drugs was determined. Antibiotics selected by the bacteriological test were administered to patients who fulfilled the criteria for sepsis. Antibiotic therapy was given until there was no evidence of intra-abdominal, wound infection, or persistent signs of sepsis. Suture abscesses were excluded if inflammation or discharge was minimal or confined to points of penetration, and the incision healed without drainage or antibiotics. The following data were collected for each patient: age, sex, body mass index, presence of diabetes mellitus, previous abdominal surgery, previous cholecystitis attack and previous ERCP procedure, duration of surgery, length of hospital stay, gallbladder rupture, suction drain use, and postoperative infections. All data were collected on paper forms by a surgeon (ET).

### 3.1. Statistical Analysis

Statistical analyses were performed with the SPSS software (SPSS: An IBM Company, version 9.0, IBM Corporation, Armonk, New York, USA). For continuous variables, normality test was use by Kolmogorov-Smirnov. The two groups were compared using the t test for normal continuous variables and the Mann-Whitney U test for non-normal continuous variables. The chi-square test for categorical variables was used. For continuous variables, numeric values are expressed as means ± SD. If the P value was less than 0.5, the evaluation was considered statistically significant.

## 4. Results

Seventy-five male and 194 female patients (mean age, 47.8 years) were enrolled in the control group and 70 male and 208 female patients were enrolled in the CEF group (mean age, 51.9 years). There was no statistically significant difference between the 2 groups in terms of sex, age, diabetes mellitus, previous abdominal surgery, body mass index, previous cholecystitis attacks and/or previous ERCP procedures, length of stay, duration of operation, intraoperative gallbladder rupture, and use of a suction drain (Table 1). 

**Table 1.** Characteristics of Patients and Surgical Outcomes

Characteristics	CEF Group, n = 278	Control Group, n = 269	P value
**Sex, No (%)**			0.47
Male	70 (25.2)	75 (27.9)	
Female	208 (74.8)	194 (72.1)	
**Diabetes mellitus, No, (%)**			0.60
Yes	38 (13.7)	41 (15.2)	
No	240 (86.3)	228 (84.8)	
**Previous abdominal surgery, No (%)**			0.71
Yes	36 (12.9)	32 (11.9)	
No	242 (87.1)	237 (88.1)	
**Previous cholecystitis attack and/or previous ERCP procedure, No (%)** ^**a**^ ** procedure, No (%)**			0.59
Yes	61 (21.9)	54 (20.1)	
No	217 (78.1)	215 (79.9)	
**Rupture of gallbladder, No (%)**			0.77
Yes	48 (17.3)	49 (18.2)	
No	230 (82.7)	220 (81.8)	
**Suction drain, No (%)**			0.79
Yes	50 (18)	46 (17.1)	
No	228 (82)	223 (82.9)	
**Postoperative infection, No (%)**			0.44
Yes	4 (1.4)	2 (0.7)	
No	274 (98.6)	267 (99.3)	
**Age, yr, Mean ± SD**	51.9 ± 13.1	47.8 ± 13.4	0.27
**Body mass index, kg/m, Mean ± SD** ^**2**^ **, Mean ± SD**	29.1 ± 4.9	28.7 ± 5.9	0.45
**Duration of operation, min, Mean ± SD**	69.5 ± 19.4	65.9 ± 17.9	0.74^b^
**Length of stay, day, Mean ± SD**	1.5 ± 0.8	1.6 ± 0.7	0.29 ^b^

^a^Abbreviations: ERCP, endoscopic retrograde cholangiopancreatography

^b^ Non-parametric test were used

Postoperative infection was observed in 4 patients (1.44%) in the CEF group and in 2 patients (0.74%) in the control group. Incidence of infection in patients was 1.1% for the entire study group. There was no statistically significant difference in the postoperative infection rate between the groups (P = .44). The OR (odds ratio) was found to be 0,513 (95% CI: 0,093-2,824). Prophylactic antibiotics did not correlate with postoperative infection. All observed infections were surgical site infections. No other postoperative systemic infectious complications (eg, sepsis, pneumonia, or urinary tract infection) were found in either group. The risk of infectious complications increased in patients with a previous attack of acute cholecystitis and/or previous endoscopic retrograde cholangiopancreatography procedure (P < 0.001), as well as in patients with a ruptured gallbladder and suction drainage use (P < 0.001 and < 0.001) (Table 2). 

**Table 2.** Risk Factors for Infectious Complications

Characteristics	Infectious Complications, n = 6	No Complication, n = 541	P value
**Sex, No (%)**			0.19
Male	3 (50)	142 (26.2)	
Female	3 (50)	399 (73.8)	
**Diabetes mellitus, No (%)**			0.87
Yes	1 (16.7)	78 (14.4)	
No	5 (83.3)	463 (85.6)	
**Previous abdominal surgery, No (%)**			0.11
Yes	2 (66.7)	64 (11.8)	
No	4 (33.3)	477 (88.2)	
**Previous cholecystitis attack and/or previous ERCP procedure, No (%)** ^**a**^ ** procedure, No (%)**			< 0.001
Yes	5 (83.3)	110 (20.3)	
No	1 (16.7)	431 (79.7)	
**Duration of operation (min), Mean ± SD**	66.7 ± 10.3	66.5 ± 25.7	0.98 ^b^
**Rupture of gallbladder, No (%)**			< 0.001
Yes	5 (83.3)	92 (17)	
No	1 (16.7)	449 (83)	
**Suction drain, No (%)**			< 0.001
Yes	5 (83.3)	91 (16.8)	
No	1 (16.7)	450 (83.2)	
**Age, yr, Mean ± SD**	47.3 ± 10.5	50 ± 13.4	0.62
**Body mass index, kg/m, Mean ± SD** ^**2**^ **, Mean ± SD**	29.5 ± 2.8	28.9 ± 5.4	0.81
**Duration of operation, min , Mean ± SD** ^**b**^ **, Mean ± SD**	66.7 ± 10.3	66.5 ± 25.7	0.98 ^b^
**Length of stay, day, Mean ± SD**	1.33 ± 0.51	1.51 ± 0.75	0.55 ^b^

^a^ Abbreviations: ERCP, endoscopic retrograde cholangiopancreatography

^b^ Non-parametric test were used

## 5. Discussion

Antibiotic prophylaxis is considered the standard protocol in open cholecystectomy as the means for reducing the incidence of infectious complications, however its use is debated in LC. It is generally recommended that a single dose of cephalosporin be administered intravenously on anesthesia induction or just before incision in a clean or clean-contaminated procedure (14). Cefazolin has been recommended for patients undergoing open cholecystectomy and other biliary surgery (12, 13). However the aim of antimicrobial prophylaxis is not to completely eradicate microorganisms from the tissue, but to reduce the number of microorganisms to such an extent that the defense mechanism of the host can effectively prevent infection by the contaminating microorganisms (15). Whether or not antibiotic prophylaxis has any effect on the occurrence of postoperative infections in LC remains controversial (1-7, 9-11). Several studies conclude that the use of prophylactic antibiotics in LC leads to a significant decrease in infectious complications (9, 10, 16). Conversely, many prospective studies suggest that antibiotic prophylaxis is probably not required in elective LC, because the infection rate of LC is already low and the use of prophylactic antibiotics does not decrease the incidence of postoperative infectious complications (1, 3, 5, 8).

The results of 5 large meta-analyses from multiple centers showed that the average rate of wound infection was 0.4% to 1.1% (2, 6, 7, 17, 18). Our data show that the incidence of infection in patients was 1.1% for the total study group, 1.44% for the CEF group, and 0.74% for the CTRL group. Similar to previous studies, there were no significant differences in infection rate between groups. Perforation during gallbladder surgery is attributed to traction, grasping, dissection, and removal of the gallbladder and occurs in 11% to 35% of LC (3, 5, 8, 10). In this study, rupture of the gallbladder occurred in 17.3% and 18.2% of CEF and CTRL patients. Many reports also have indicated that wound infections are not related to bile culture, rupture of gallbladders, or spillage of gallbladder stones or bile (1, 19). However, similar to some other studies, our study also demonstrated that rupture of the gallbladder and bile spillages contribute to increased rates of postoperative infection (20). Some studies have reported that the use of drains increases the occurrence of fluid in the sub-hepatic space after LC and drain use increases infection rates owing to a foreign body reaction (20). In this study, the postoperative infection rate was significantly higher in patients in whom drains were used compared with those in whom drains were not used. We think that the increased infection rate in patients in whom drains were used may be related to a more complicated course (gall bladder perforation, bleeding from the bile tree etc.) of those patients.

Some studies (1-3) have recommended that antimicrobial prophylaxis be given only to patients with high-risk features (e.g. old age, diabetes mellitus, or episodes of colic within 30 days of surgery). Similar with those studies, our study showed that the rate of postoperative infection was significantly higher in patients with a previous attack of cholecystitis and/or a previous endoscopic retrograde cholangiopancreatography procedure. However, unlike some other studies (5), we found no correlation between postoperative infection rate and age or presence of diabetes mellitus. Our study was conducted on a low-risk patient group. We believe that studies with a larger sample size should be performed on this subject in the future. In conclusion, we found no correlation between cefazolin prophylaxis and postoperative infectious complications in ASA I-II and elective LC patients. However, in our opinion one must watch for risk of intraoperative infection in patients with a previous attack of cholecystitis and/or previous endoscopic retrograde cholangiopancreatography procedure, and for risk of postoperative infections in patients with rupture of gallbladder and suction drain use.

## References

[1] Koc M, Zulfikaroglu B, Kece C, Ozalp N (2003). A prospective randomized study of prophylactic antibiotics in elective laparoscopic cholecystectomy.. Surg Endosc..

[2] McGuckin M, Shea JA, Schwartz JS (1999). Infection and antimicrobial use in laparoscopic cholecystectomy.. Infect Control Hosp Epidemiol..

[3] Tocchi A, Lepre L, Costa G, Liotta G, Mazzoni G, Maggiolini F (2000). The need for antibiotic prophylaxis in elective laparoscopic cholecystectomy: a prospective randomized study.. Arch Surg..

[4] Meijer WS, Schmitz PI, Jeekel J (1990). Meta-analysis of randomized, controlled clinical trials of antibiotic prophylaxis in biliary tract surgery.. Br J Surg..

[5] Chang WT, Lee KT, Chuang SC, Wang SN, Kuo KK, Chen JS (2006). The impact of prophylactic antibiotics on postoperative infection complication in elective laparoscopic cholecystectomy: a prospective randomized study.. Am J Surg..

[6] Yan RC, Shen SQ, Chen ZB, Lin FS, Riley J (2011). The role of prophylactic antibiotics in laparoscopic cholecystectomy in preventing postoperative infection: a meta-analysis.. J Laparoendosc Adv Surg Tech A..

[7] Sanabria A, Dominguez LC, Valdivieso E, Gomez G (2010). Antibiotic prophylaxis for patients undergoing elective laparoscopic cholecystectomy.. Cochrane Database Syst Rev..

[8] Uludag M, Yetkin G, Citgez B (2009). The role of prophylactic antibiotics in elective laparoscopic cholecystectomy.. JSLS..

[9] Shindholimath VV, Seenu V, Parshad R, Chaudhry R, Kumar A (2003). Factors influencing wound infection following laparoscopic cholecystectomy.. Trop Gastroenterol..

[10] Uchiyama K, Kawai M, Onishi H, Tani M, Kinoshita H, Ueno M (2003). Preoperative antimicrobial administration for prevention of postoperative infection in patients with laparoscopic cholecystectomy.. Dig Dis Sci..

[11] Mir MA, Malik UY, Wani H, Bali BS (2013). Prevalence, pattern, sensitivity and resistance to antibiotics of different bacteria isolated from port site infection in low risk patients after elective laparoscopic cholecystectomy for symptomatic cholelithiasis at tertiary care hospital of Kashmir.. Int Wound J..

[12] Westphal JF, Brogard JM (1999). Biliary tract infections: a guide to drug treatment.. Drugs..

[13] Munro R, Sorrell TC (1986). Biliary sepsis. Reviewing treatment options.. Drugs..

[14] Nichols RL (2001). Preventing surgical site infections: a surgeon's perspective.. Emerg Infect Dis..

[15] Page CP, Bohnen JM, Fletcher JR, McManus AT, Solomkin JS, Wittmann DH (1993). Antimicrobial prophylaxis for surgical wounds. Guidelines for clinical care.. Arch Surg..

[16] Dervisoglou A, Tsiodras S, Kanellakopoulou K, Pinis S, Galanakis N, Pierakakis S (2006). The value of chemoprophylaxis against Enterococcus species in elective cholecystectomy: a randomized study of cefuroxime vs ampicillin-sulbactam.. Arch Surg..

[17] Shea JA, Berlin JA, Bachwich DR, Staroscik RN, Malet PF, McGuckin M (1998). Indications for and outcomes of cholecystectomy: a comparison of the pre and postlaparoscopic eras.. Ann Surg..

[18] Shea JA, Healey MJ, Berlin JA, Clarke JR, Malet PF, Staroscik RN (1996). Mortality and complications associated with laparoscopic cholecystectomy. A meta-analysis.. Ann Surg..

[19] Gold-Deutch R, Mashiach R, Boldur I, Ferszt M, Negri M, Halperin Z (1996). How does infected bile affect the postoperative course of patients undergoing laparoscopic cholecystectomy?. Am J Surg..

[20] Suh SW, Park JM, Lee SE, Choi YS (2012). Accidental gallbladder perforation during laparoscopic cholecystectomy: does it have an effect on the clinical outcomes?. J Laparoendosc Adv Surg Tech A..

